# Supplementary figures and images for: Can nighttime lights serve as a proxy for economic inequality at the local administrative unit scale? Evidence from Spain
Source: PLoS One. 2025 Dec 10;20(12):e0319890. doi: 10.1371/journal.pone.0319890 (PMC12694823; doi:10.1371/journal.pone.0319890)

Number of Municipalities

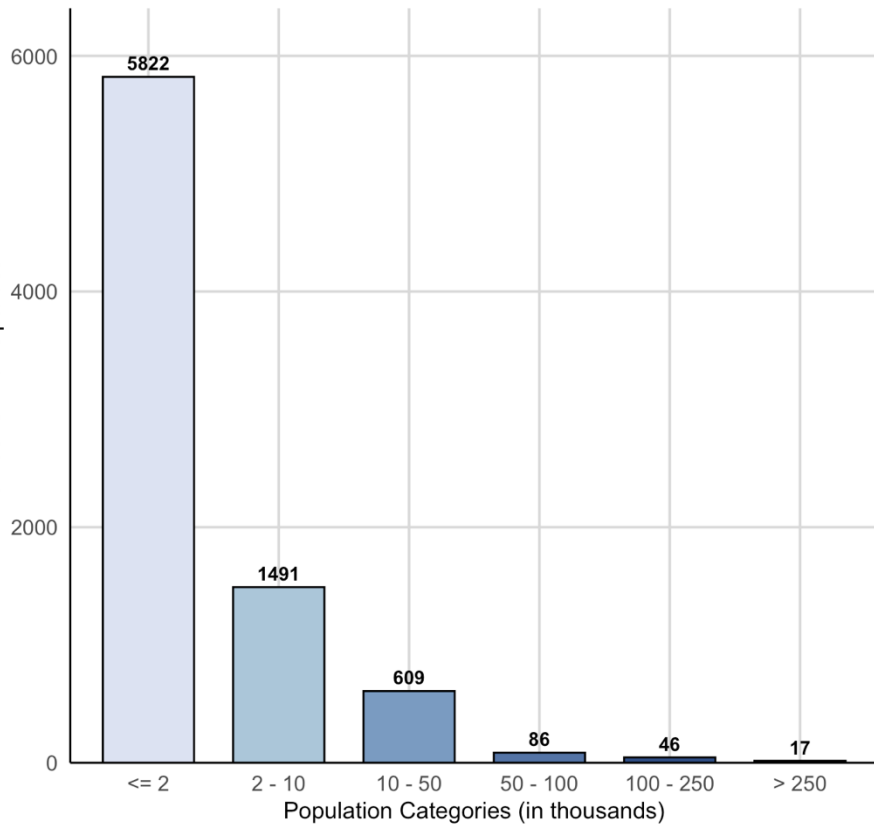

Population Categories

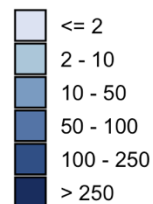

Supplement: S1 Fig — Source: Municipal register of inhabitants, National Institute of Statistics (INE). (PDF) [file pone.0319890.s001.pdf]
