# Supplementary material for: Can nighttime lights serve as a proxy for economic inequality at the local administrative unit scale? Evidence from Spain
Source: PLoS One. 2025 Dec 10;20(12):e0319890. doi: 10.1371/journal.pone.0319890 (PMC12694823; doi:10.1371/journal.pone.0319890)
Supplement: S2 Fig — Each Gini estimate, except the National Gini (calculated from national-level income data), is derived from the average values across all Spanish municipalities. Source: Atlas of Household Income Distribution (ADRH)38 produced by the National Statistics Institute of Spain (INE). (PDF) [file pone.0319890.s002.pdf]

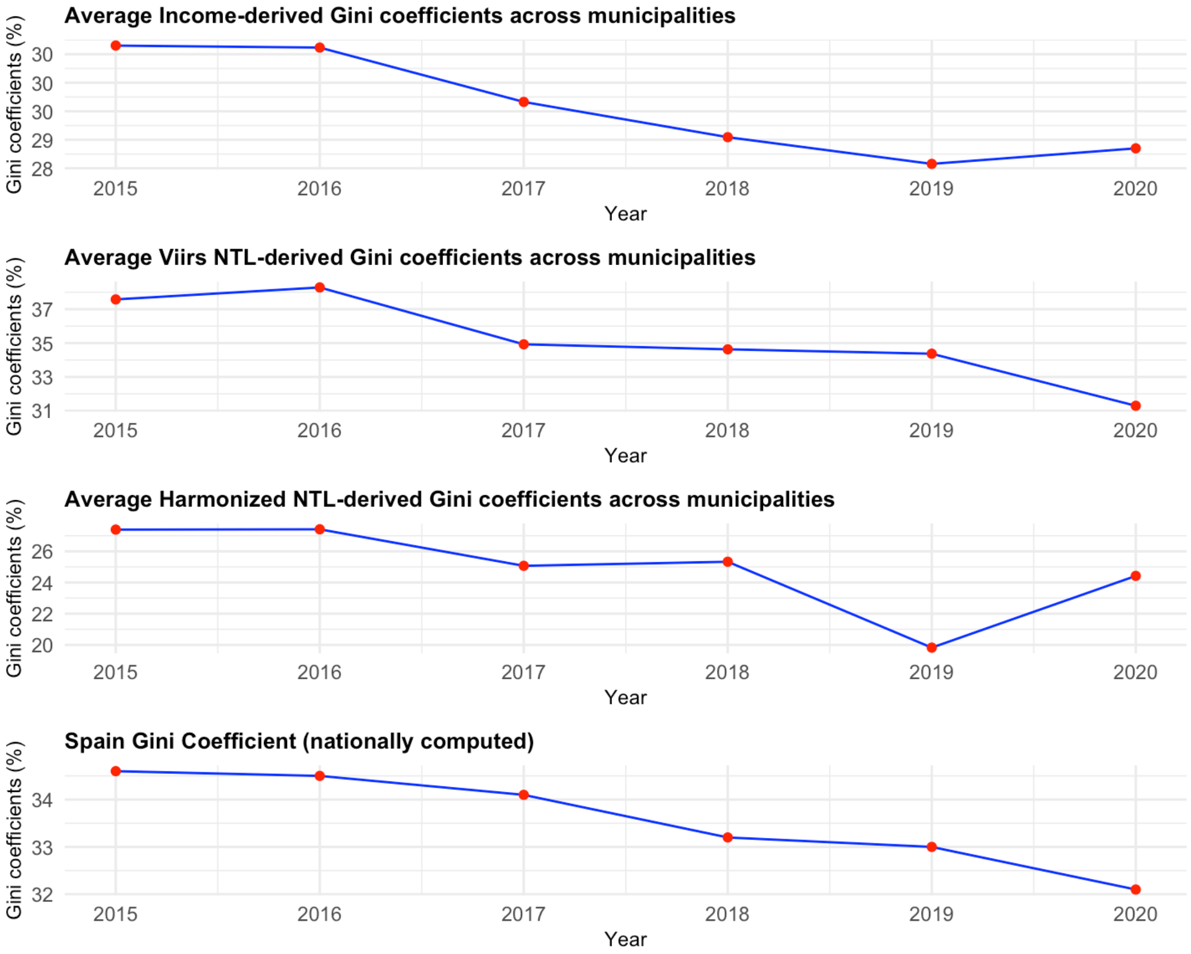

Average Income-derived Gini coefficients across municipalities
Gini coefficients (%)
30
30
30
29
28
2015
2016
2017
2018
2019
2020
Year
Average Viirs NTL-derived Gini coefficients across municipalities
Gini coefficients (%)
37
35
33
31
2015
2016
2017
2018
2019
2020
Year
Average Harmonized NTL-derived Gini coefficients across municipalities
Gini coefficients (%)
26
24
22
20
2015
2016
2017
2018
2019
2020
Year
Spain Gini Coefficient (nationally computed)
Gini coefficients (%)
34
33
32
2015
2016
2017
2018
2019
2020
Year
