# Supplementary material for: Can nighttime lights serve as a proxy for economic inequality at the local administrative unit scale? Evidence from Spain
Source: PLoS One. 2025 Dec 10;20(12):e0319890. doi: 10.1371/journal.pone.0319890 (PMC12694823; doi:10.1371/journal.pone.0319890)
Supplement: Attachment — Submitted filename: Response_to_Reviewers.docx [file pone.0319890.s004.docx]

# Response to Reviewers – Manuscript ID: PONE-D-25-06682

Title: Unprecedented-Scale Estimation of Spatiotemporal Changes in Economic Inequality Using Remote Sensing: Insights from Spain

Dear Editor and Reviewers,

**Dear Editor and Reviewers,**

We would like to express our sincere gratitude for your constructive and insightful comments on our manuscript. We have carefully addressed each suggestion and believe the revisions have significantly strengthened the clarity, depth, and overall quality of the paper. In the following pages, we provide detailed, point-by-point responses to all reviewer and editorial comments. We have uploaded a clean version of the revised manuscript, and a tracked-changes version.

Reviewer 1

**1. The theme is economic inequality but “economic” is not shown in the title.**

Response: We revised the title to: “Unprecedented-Scale Estimation of Spatiotemporal Changes in Economic Inequality Using Remote Sensing: Insights from Spain.” The idea is to incorporate “Economic inequality” as suggested by you, while retain two concepts that are key for the paper contribution, first the unprecedented scale, second, the spatiotemporal approach. Also, the fact we use Spain as our case study.

**2. Grammar - use “remote sensing data” instead of “remotely sensed data”**

Response: We have revised the manuscript accordingly, using “remote sensing data”.

**3. Line 145–147: How is age shown?**

Response: We clarified that age is incorporated via the OECD equivalence scale used by Spain’s income statistics, which gives different weights to children and adults in income calculations. This variable refers however to the benchmark data, not light data.

**4. Homogeneity issue: WorldPop is derived partly from NTL. How do you address the potential circularity/homogeneity issue?**

Response: We added a paragraph in the discussion section to clarify this potential limitation and discussed why the impact is likely minimal due to WorldPop’s use of multiple auxiliary variables (e.g., building footprints, land cover, etc.), not solely NTL.

**5. The conclusion and influence mechanism are not shown**

Response: We expanded the discussion sections to include possible mechanisms linking light distribution and inequality (e.g., uneven infrastructure development, wealth clustering). Also, the discussion section was restructure to follow this logic: Restatement of the research problem, Summary of the main findings, Interpretation of the results, Comparison with previous research, Explanation of any unexpected findings or discrepancies, Discussion of the implications of the results, Identification of limitations and suggestions for prospective research. Although I did not add that subheadings, this is the implicit structure.

A new section on conclusions was also added, following this estructure: Restatement of the research question, Summary of the main findings, Discussion of the implications of the research and Suggestions for future research. In this shorter.

**6. Theoretical link: How does light reflect inequality?**

Response: We included new text in discussion explaining the theoretical link between light intensity, and the need to further explore that relation in the future in more qualitative studies. For instance, how stronger light intensity relates to standard of living in situ.

**7. How were the negative NTL values removed?**

Response: We clarified this in the methods section: negative values were excluded implicitly during rasterization and aggregation in R using na.rm = TRUE and filtering of non-positive values. This step is now clearly described in the updated manuscript.

**8. Rural informal economy not quantified**

Response: we now explain that the differences find between NTL based Gini and Income based Gini in areas with high unemployment and informal economy could indicate that NTL Gini is unrevealing aspects not covered by official statistics. However, we emphasize that this is just an observation (visual) that requires further investigation in the future.

**9. Low R² – discuss**

Response: We added a paragraph in the discussion acknowledging the low R² and offering several explanations. Specifically, we argue that cross-sectional models likely underperform because light intensity reflects not only income but also structural factors like infrastructure, energy use, and urban form, which vary across municipalities. In contrast, panel models benefit from temporal stability in these factors, allowing NTL changes to better reflect true economic shifts. This suggests lights-per-capita serve better as indicators of change over time rather than absolute inequality, especially where conventional data are limited.

**10. Expand explanation for Figs 2 and 3**

Response: Both captions have been substantially expanded for clarity. See revised captions in the manuscript. We have also added additional comments on the figures in the text.

**11. Add more literature using remote sensing for economy/population**

Response: We have added 15+ new references.

**Reviewer 2**

**1. Abstract needs to be more focused**

Response: We streamlined the abstract into four concise elements (background, methods, results, significance), emphasizing NTL’s potential as a scalable inequality proxy.

**2. Broader theoretical context: urbanization, land use, policy**

Response: the discussion now include a further discussion on how future research on the correlation between lights derived from remote sensing and official statistics could benefit from incorporating urban structure and land use related variables.

**3. Low R² in Table 1 – explain**

Response: We added a paragraph in the discussion acknowledging the low R² and offering several explanations. Specifically, we argue that cross-sectional models likely underperform because light intensity reflects not only income but also structural factors like infrastructure, energy use, and urban form, which vary across municipalities. In contrast, panel models benefit from temporal stability in these factors, allowing NTL changes to better reflect true economic shifts. This suggests lights-per-capita serve better as indicators of change over time rather than absolute inequality, especially where conventional data are limited.

**4. Harmonized vs VIIRS – add pros/cons**

Response: We added a paragraph comparing both NTL products and outlined scenarios where each is preferable (e.g., long-term trends vs. finer spatial resolution). Each of the sources are also described in the methodological section.

**5. Informal economy – how to test this?**

Response: we now explain that the differences find between NTL based Gini and Income based Gini in areas with high unemployment and informal economy could indicate that NTL Gini is unrevealing aspects not covered by official statistics. However, we emphasize that this is just an observation (visual) that requires further investigation in the future. We also added a comment in the discussion suggesting that future research should be conducted in a more qualitative way (for instance ethnography) to better understand such aspects as informal economy.

**6. Future research: other geospatial layers**

Response: We included this in the “Future Directions” section, noting how adding urban density, infrastructure maps, or land use data could enhance inequality estimation.

**7. Add more references**

Response: We added over 15 new references to support this work.

Please don’t hesitate to let us know if further changes are needed. We thank you again for the opportunity to revise our work and for the constructive guidance received.

**Sincerely,**
Dr. Xaquín S. Pérez-Sindín

On behalf of all co-authors

Faculty of Economics, University of Warsaw

Email: xperez@wne.uw.edu.pl
