# Supplementary material for: Can nighttime lights serve as a proxy for economic inequality at the local administrative unit scale? Evidence from Spain
Source: PLoS One. 2025 Dec 10;20(12):e0319890. doi: 10.1371/journal.pone.0319890 (PMC12694823; doi:10.1371/journal.pone.0319890)
Supplement: Attachment — Submitted filename: Response to Reviewers.docx [file pone.0319890.s005.docx]

Response to Reviewers – Manuscript ID: PONE-D-25-06682

Dear Editor and Reviewers,

We thank you for your constructive and insightful feedback. We have addressed each comment carefully, and believe the revisions have improved the clarity, depth, and overall quality of the paper. Below, we provide detailed point-by-point responses, along with both a clean and a tracked-changes version of the revised manuscript.

Response to Reviewer 1’s Comments

1. **Title**

We have updated the title to

**“Can Nighttime Lights Serve as a Proxy for Economic Inequality at the Local Administrative Unit Scale? Evidence from Spain”**

Which we believe better reflect the aim and scope of the article.

**2. Abstract length**

Thank you for pointing this out. The abstract has been reduced to fewer than 350 words while retaining the essential elements of background, methods, results, and implications.

**3. Keywords**

We appreciate your suggestion and have updated the keywords to include: spatiotemporal characteristic, nighttime lights, economic inequality, local administrative unit, remote sensing, Spain, inequality mapping.

**4. Specific time reference**

We have revised vague temporal expressions to be more specific. For example:

“Over the past two decades” instead of “In recent years”

**5. Number of references**

We have reduced the number of references per sentence to no more than three, following your recommendation.

**6. NTL and income vs GDP**

Our study evaluates the capacity of an NTL-based Gini coefficient to predict the conventional tax-statistics-based Gini for measuring economic inequality. The focus is therefore inequality, making income the appropriate benchmark rather than GDP. Literature in this specific subfield is limited, and the unresolved correlation between NTL- and income-based inequality measures is central to our paper. We have retained the referenced paragraphs in the Introduction as they justify Spain as a case study and outline our novel coefficient estimation, which the Methods section then develops in detail.

**7. Methods structure**

We have reorganized the Methods section into three subsections: Study Area, Data Sources, and Methods. We retained concise descriptions of each dataset because their limitations are important for assessing the reliability of our results. However, we have reduced unnecessary detail.

**8 Scale**

We have emphasized in the title, abstract, and text that our analysis is at the local administrative unit (municipality) scale, using indicators of inequality calculated based on all pixels within each municipality.

**9. The worldpop data is population density, how to transfer number of populations, it should be illustrated.**

The worldpop data is actually population count. We have now indicated it clearly in the text.

**10. Control variables and phenomenon explanation**

The aim of this study is to assess whether nighttime-light-based Gini coefficients correlate with those derived from tax statistics. While existing literature has consistently shown a strong relationship between NTL and economic activity, only a few studies—those to which we seek to contribute—have examined whether NTL can also predict inequality. Using Spain as a case study and working at an unprecedented level of detail (municipality rather than regional scale), our objective is to produce a validated indicator of inequality for each municipality.

**11. Focus of the study**

We agree that urban–rural disparities are not a central focus and have removed them from the keywords. The manuscript now emphasizes the main contribution: validating NTL as a proxy for inequality and comparing VIIRS and Harmonized datasets.

**12. Limitations**

We appreciate the reviewer’s observation regarding the number of limitations listed. In the revised manuscript, we have streamlined the “Identification of Limitations” section to focus on the most relevant constraints of our approach and to distinguish between those that could be addressed in future work and those inherent to the methodology.

Specifically, we removed the limitation related to potential noise from commercial activity and other non-residential lighting sources, as this issue is already addressed in the Methods section through the exclusion of all pixels without population counts. This preprocessing step effectively mitigates the bias from uninhabited but brightly lit areas.

We also removed the point on accounting for spatial autocorrelation. While such modelling refinements can be valuable in some contexts, their contribution to the main objective of this study—validating nighttime lights as a proxy for inequality—is uncertain. For prudence and clarity, we decided not to introduce methodological extensions that may not directly serve our primary research goal.

The final limitations section now emphasizes four key issues: (1) the role of WorldPop in population estimation, (2) sensor-related biases, (3) known biases in administrative income data, and (4) the conceptual limitations of lights-per-capita as a socioeconomic indicator. For each, we note whether and how it could be addressed in future research. We believe this revised, more focused discussion better addresses the reviewer’s concern while preserving transparency.
