# Supplementary material for: Can nighttime lights serve as a proxy for economic inequality at the local administrative unit scale? Evidence from Spain
Source: PLoS One. 2025 Dec 10;20(12):e0319890. doi: 10.1371/journal.pone.0319890 (PMC12694823; doi:10.1371/journal.pone.0319890)
Supplement: Attachment — Submitted filename: Response_to_Reviewers_auresp_3.docx [file pone.0319890.s006.docx]

Response to Reviewers – Manuscript ID: PONE-D-25-06682

Dear Editor and Reviewers,

We thank you once again for your constructive feedback. We have carefully addressed all remaining points raised in the latest round of review. The abstract has been shortened to under 300 words, vague temporal phrasing has been replaced with specific years, excessive citations have been reduced, the implications of regression models have been clarified, missing dataset websites have been added, and the issue of WorldPop’s reliance on nighttime lights has been further explained in the discussion.

We believe these final revisions resolve the concerns and improve the manuscript’s clarity and precision. Below, we provide detailed point-by-point responses.

Response to Reviewer 1’s Comments

1. Abstract length

We thank the reviewer for this observation. The abstract has now been significantly shortened to under 300 words, following the PLOS ONE guidelines. Redundant phrasing and descriptive details (such as Spain’s relative inequality and geographic diversity) were removed or streamlined to improve conciseness and focus.

2. Time specification (“Over the past two decades”)

We agree with the reviewer that the original phrasing was too vague. This has been revised to: “Following the early 2000s financial liberalization and the 2008 global financial crisis…”, which provides a more precise temporal anchor.

3. Excessive citations in one sentence

We acknowledge the reviewer’s point and have reduced the number of references per sentence to 4–5, except in one case ([25–32]) where all cited sources are essential.

4. Cross-sectional vs. panel regression implications

We appreciate this request for clarification. The implications of similarities and differences between the cross-sectional and panel models are now explicitly highlighted in both the abstract and the conclusions. In particular, we emphasize that panel models capture temporal dynamics more effectively, while cross-sectional regressions highlight spatial patterns at given points in time.

5. Data sources and missing website links

We thank the reviewer for pointing this out. All datasets now include complete references, with URLs/websites added where they were previously missing.

6. WorldPop dependence on nighttime lights

We acknowledge this concern and reiterate that it was addressed in the revised discussion. We clarified that while NTL data are included among the covariates in the WorldPop modelling process, their role is minor and supportive compared to census, land cover, infrastructure, and accessibility data. We also added that alternative population datasets (solely derived from census or surveys) could be used in future research to test robustness.
